# Measuring upper limb function and patient reported outcomes after major breast cancer surgery: a pilot study in an Asian cohort

**DOI:** 10.1186/s12893-020-00773-0

**Published:** 2020-05-19

**Authors:** Kai Siang Chan, Ding Zeng, Joelle Hoi Ting Leung, Belinda Si Yin Ooi, Kit Teng Kong, Yi Heng Yeo, Jerry Tiong Thye Goo, Clement Luck Khng Chia

**Affiliations:** 1 MOH Holdings Pte Ltd, Singapore, Singapore; 2 Lee Kong Chian School of Medicine, Nanyang Technological University, Singapore, Singapore; 3 Department of General Surgery, Khoo Teck Puat Hospital, Singapore, Singapore; 4 Department of Physiotherapy, Khoo Teck Puat Hospital, Singapore, Singapore

**Keywords:** Breast surgery, Rehabilitation, Upper limb function

## Abstract

**Background:**

Breast cancer is the most common cancer in women worldwide. Major breast cancer surgery especially with axillary lymph node dissection (ALND), is associated with upper limb functional decline. Majority of studies are conducted in Western population and may not be applicable to Asians. This pilot study aims to evaluate whether major breast surgery results in upper limb functional impairment in a cohort of Asian women with breast cancer.

**Methods:**

This is a prospective cohort study of 41 patients who underwent 44 major breast surgeries from April 2018 to August 2019. Main inclusion criteria were patients over 21 years of age undergoing major breast surgery for breast cancer. Major breast surgery was defined as wide local excision (WLE) or mastectomy. Main exclusion criteria were patients with pre-existing neurological or rheumatological co-morbidities affecting upper limb function or previous trauma with resulting deformities to the upper limbs. Patients underwent early rehabilitation from post-operative day 1. Shoulder flexion and abduction active range of motion (AROM) and QuickDASH disability score were assessed 1 week before surgery, post-operative week 2 and week 6. Baseline demographics and peri-operative data were also collected.

**Results:**

Median age was 62.5 years. There were 16 (36.4%) wide local excisions and 28 (63.6%) simple mastectomies. Two (4.5%) cases had neoadjuvant chemotherapy. Fifteen (34.1%) cases had ALND. At post-operative week 6, shoulder flexion was comparable to baseline (*p* = 0.775), while abduction improved from baseline (*p* = 0.016). However, QuickDASH disability score was significantly worse at post-operative week 6 compared to baseline (median score 2.5 vs 0, *p* = 0.027). Subgroup analysis of patients with ALND demonstrated significantly worse QuickDASH disability score at post-operative week 6 (*p* = 0.010) but not for patients with only sentinel lymph node biopsy (*p* = 0.396).

**Conclusion:**

This pilot study in an Asian cohort found that patients were able to regain AROM of shoulder after major breast surgery at post-operative week 6 but had a worse QuickDASH disability score, especially in the subgroup with ALND. Aggressive and early rehabilitation should be encouraged. However, a longer follow-up is required to evaluate long term functional outcomes.

## Background

Breast cancer is the most common cancer and leading cause of death in women worldwide with an incidence of 24.2% in all diagnosed cancers and a mortality of 15.0% in 2018 [1]. It is also the most common cancer locally in Singapore, occurring in almost 1 in 3 diagnosed cancers in females [2]. Surgical options for breast cancer include breast-conserving surgery and mastectomy. When diagnosed in early stage, breast cancer surgery offers excellent oncological outcomes with a 5-year survival of up to 92.9% [3].

The role of rehabilitation and functional outcomes are however less studied in breast cancer surgery despite musculoskeletal complications such as pain, decreased joint mobility and reduced muscle strength being commonly reported after major breast surgery [4–6]. Metrics such as function, quality of life, psychosocial impact and satisfaction are also generally not assessed by many breast units in the world. A systematic review by Hidding et al. demonstrated significant upper limb functional impairments such as reduced range of motion and activities in daily living on long-term follow-up [7]. In addition, axillary lymph node dissection (ALND) has been demonstrated to be an additional risk factor for upper limb functional impairment [7].

Functional outcomes are of increasing importance in breast cancer as survival and prognosis for breast cancer is excellent and improving over the years [3]. With more than half of diagnosed breast cancers occurring in middle-aged women between the ages of 45 and 64 years [2], it is pertinent to ensure preservation of upper limb function as this group of patients may be the main caregivers of their families, engage in an active lifestyle and at the peak of their careers.

Several studies have discussed about the importance of rehabilitation post-surgery to achieve upper limb functional recovery [8–10]. It has been also suggested that early rehabilitation may reduce the onset of complications [11]. Most of the studies were performed in the Western population, but studies in the East have shown that Asians may differ with higher body fat percentage, lower bone mass and lower level of physical activity [12–14]. These factors may influence the functional outcomes in patients with breast cancer. This study aims to evaluate whether major breast surgery results in functional impairment in a cohort of Asian women with breast cancer.

## Methods

**Fig. 1:**
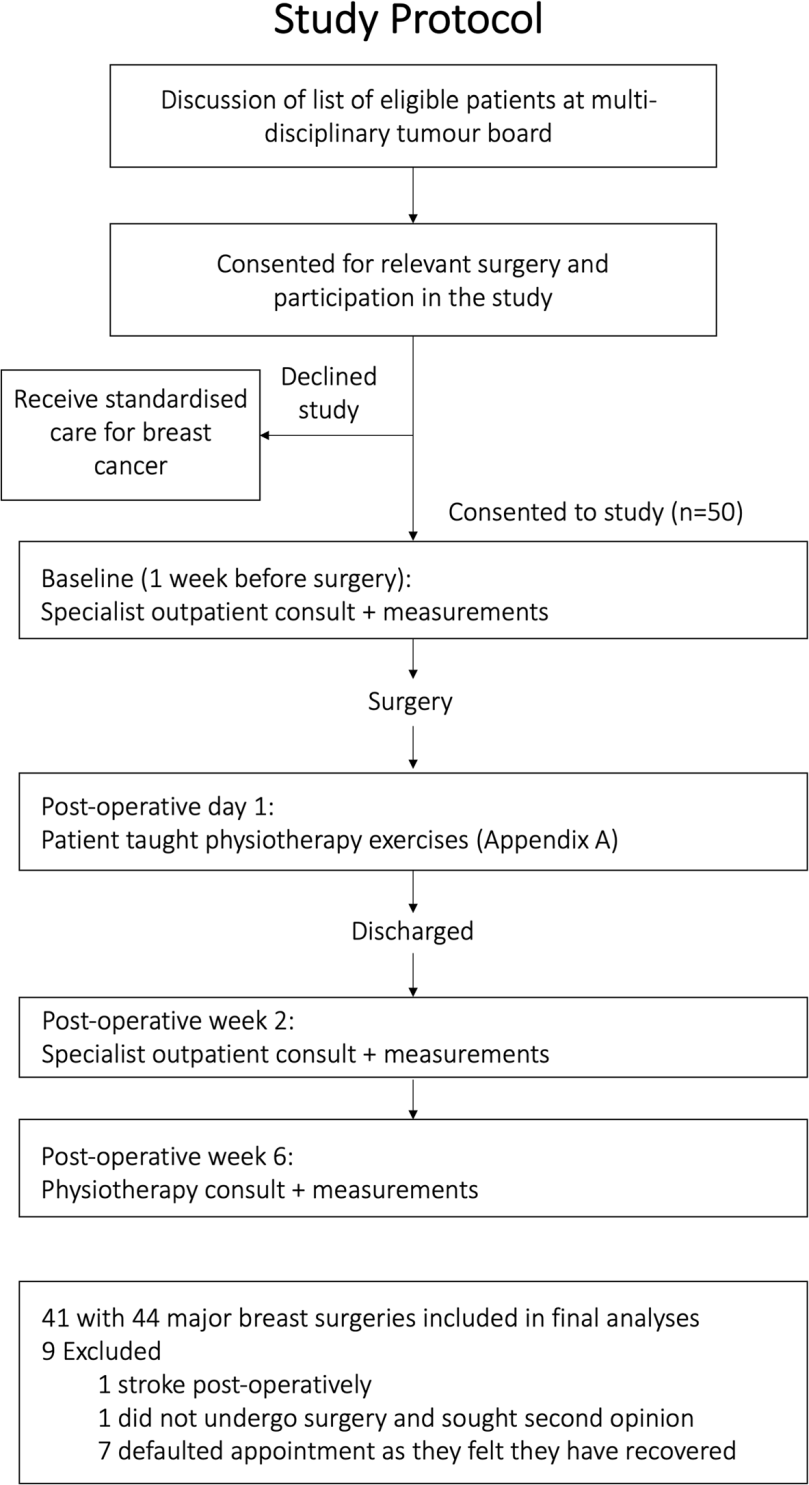
Study protocol outlining the conduct of this research study

This is a single center prospective cohort study conducted at a university-affiliated teaching hospital. Patients were recruited between April 2018 to August 2019 with a follow-up of 6 weeks post-surgery. Main inclusion criteria were patients who were over 21 years of age undergoing major breast surgery for breast cancer. Major breast surgery was defined as wide local excision (WLE) or mastectomy performed for patients with diagnosed breast cancer. Main exclusion criteria were patients with pre-existing neurological or rheumatological co-morbidities affecting upper limb function or previous trauma with resulting deformities to the upper limbs. Patients who underwent mastectomy with reconstruction were also excluded. A total of 50 patients were recruited for this study and 9 patients were excluded from the final analysis (Fig. 1). Reasons for exclusion were described in Fig. 1. This study was approved by a local institutional review board (Ref: 2018/00156). All patients involved in the study provided written informed consent. The study protocol is shown in Fig. 1.

Patients were assessed in our outpatient one-stop breast clinic and underwent triple assessment with (1) clinical assessment, (2) radiological assessment with mammogram and breast ultrasound and (3) pre-operative biopsy. Patients with breast cancer confirmed histologically were staged and discussed in the multi-disciplinary breast tumour board prior to surgery. Informed consent was taken by the surgeons in the breast specialist outpatient clinic for participation in the study. All patients were placed on standardised post-operative care pathway. Patients who underwent WLE were discharged as day surgery, while patients who underwent mastectomy were discharged on post-operative day 1. Patients who received mastectomy were discharged with a drain placed at the chest wall that was removed in the outpatient clinic within a week. Patients who had axillary clearance had additional drain placed in the axilla which was similarly removed within a week. Post-operative analgesia was standardised with paracetamol and non-steroidal inflammatory drugs for a duration of 1 week.

Upper limb function was assessed via measurement of shoulder flexion and abduction active range of motion (AROM) using a goniometer (12″ Protractor Goniometer, Model 64, Prestige Medical, USA) (Fig. A.1, Table A.1) and completion of the QuickDASH questionnaire (Table B.1). The QuickDASH questionnaire is a quick 11-point questionnaire which assesses subjective physical function and extent of symptoms [15]. The QuickDASH disability score was calculated upon completion of the questionnaire. Upper limb AROM measurements were performed by a dedicated breast care nurse and physiotherapist with formal standardization training session (Fig. A.1, Table A.1) conducted for both individuals prior to the start of the study.

Shoulder AROM and QuickDASH scores were assessed three times during the course of the study: (1) one week prior to surgery to assess patient’s baseline function in the specialist outpatient clinic, (2) 2 weeks post-operative in the follow-up outpatient clinic and (3) 6 weeks post-operative at the physiotherapy clinic.

### Rehabilitation regime

Patients were taught and prescribed a standardized set of shoulder and upper limb exercises immediately post-surgery or on post-operative day 1 prior to discharge (Table C.1) and were instructed to perform the exercises daily at home. Patients were assessed for compliance to the rehabilitation programme at 2 weeks and reiterations of exercises were made. The detailed post-rehabilitation protocol is shown in Table C.2.

### Surgical technique of axillary lymph node dissection

The patient was positioned supine with her arms extended on an arm board at ≤90 degrees abduction from the chest wall.

Axillary lymph node dissection was performed through the same incision for patients with modified radical mastectomy. A separate 4 cm incision was made in the axilla, two finger breadths below the hair bearing skin and perpendicular to the lateral edge of the pectoralis major for patients with WLE.

During ALND, the anatomical boundaries were first defined. The lateral edge of the pectoralis major muscle was identified and retracted medially to expose the pectoralis minor muscle and allow interpectoral dissection of Rotter’s lymph nodes. The medial pectoral neurovascular bundle was preserved during the dissection. The clavipectoral fascia was entered and axillary fat pad along lateral chest wall was dissected. Long thoracic nerve along the lateral chest wall was identified and preserved. Next, the latissimus dorsi border was identified. Dissection along the latissimus dorsi border was extended inferiorly until the muscle begun to pull toward the chest wall and superiorly to the tendon of insertion.

Dissection proceeded superiorly till the axillary vein was identified. The thoracodorsal bundle was identified and preserved before mobilization of the axillary fat pad and dissection off the chest wall and the inferior surface of the axillary vein and off the thoracodorsal bundle posteriorly. The intercostobrachial nerve was preserved if possible but small branches running through the axilla fat pad that cannot be spared were ligated.

### Data collection

Patient demographics and intra-operative records were collected from the hospital electronic medical records. Patients who defaulted any of the follow-up appointments were contacted by the study administrator via phone interview to ascertain the reason for defaulting.

### Study outcomes

Primary study outcomes were AROM of shoulder flexion and abduction and QuickDASH disability score (Table B.1). Secondary outcomes were arm pain and numbness scores derived from the QuickDASH questionnaire.

### Statistical analysis

All the data were tabulated into a Microsoft Excel sheet and transposed into SPSS version 25.0 (SPSS Inc., Chicago, III., USA) for statistical analyses. All continuous data were expressed as mean or median and analysed by Wilcoxon-signed rank test or Mann-Whitney U test. All categorical variables were described as percentage and compared by either chi-squared or Fisher’s exact test. *P*-value < 0.05 was considered statistically significant.

## Results

A total of 50 patients were recruited for this study and 9 patients were excluded from the final analysis (Fig. 1). Forty-one patients who underwent 44 major breast surgeries were included in the analysis. Three patients had bilateral major breast surgeries, of which, two had bilateral breast cancer, and one underwent a right mastectomy for breast cancer with prophylactic left mastectomy as requested by the patient. There were no nerve injuries to brachial plexus, long thoracic nerve, thoracodorsal and medial pectoral neurovascular bundle in our study. Patient demographics are summarized in Table 1. Operative and histopathological details are summarized in Table 2.

**Table 1:** Patient demographics and clinical profile

	*N* = 44 (%)
Age, median (IQR)	62.5 (53.3–65)
Gender, female	44 (100)
Co-morbidities	
Diabetes mellitus	10 (22.7)
Chronic kidney disease	2 (4.5)
Ischemic heart disease	2 (4.5)
Previous breast surgery	2 (4.5)
Clinical presentation	
Asymptomatic (detected on screening)	12 (27.3)
Breast mass	29 (65.9)
Nipple discharge	2 (4.5)
Mastalgia	2 (4.5)
Axillary lymphadenopathy (clinical)	4 (9.1)
Type of major breast surgery	
Wide local excision	16 (36.4)
With SLNB	16 (100)
With ALND	5 (31.3)
Simple mastectomy	28 (63.6)
With SLNB	27 (96.4)
With ALND	10 (35.7)
Neoadjuvant chemotherapy	4 (9.1)

*ALND* Axillary lymph node dissection, *IQR* Interquartile range, *SLNB* Sentinel lymph node biopsy

**Table 2:** Operative and histopathological details

	*N* = 44 (%)
Sentinel lymph node biopsy	43 (97.9)
Axillary lymph node dissection	15 (34.1)
Oncoplastic procedure performed during breast conserving surgery	1 (6.3)^b^
Diagnosis^a^, n (%)	
DCIS	2 (4.7)
Invasive carcinoma	41 (95.3)
Size of tumour (cm), median (IQR)	2.2 (1.5–3.5)
Seroma or haematoma	17 (38.6)

*IQR* Interquartile range

^a^One of the major breast surgeries was done prophylactically with presence of tumour only on the contralateral side, hence *n* = 43 for this case

^b^Expressed as over number of patients who underwent breast conserving surgery

**Fig. 2:**
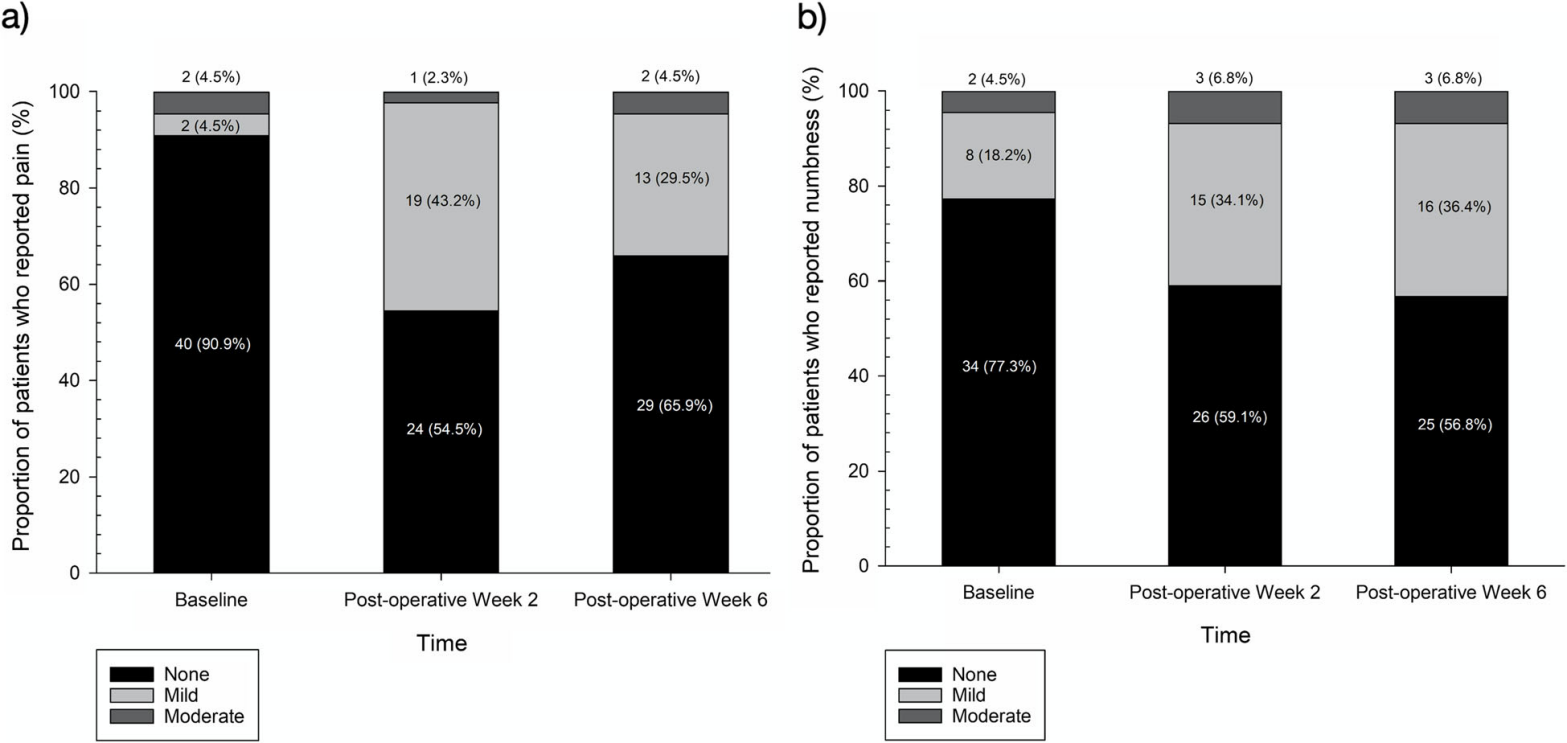
Severity of (**a**) pain and (**b**) numbness reported at baseline, post-operative week 2 and post-operative week 6

Table 3 summarizes the shoulder flexion and abduction AROM, pain score, numbness score and QuickDASH disability score of all the included cases pre-operatively, post-operative week 2 and post-operative week 6. Components of the QuickDASH disability score are shown in Table B.1 (Appendix B). Figure 2a and b represents the severity of pain and numbness reported at different time intervals of the study.

**Table 3:** Upper limb function of all patients who underwent major breast surgery

	Total (*n* = 44)				ALND (*n* = 15)				No ALND (*n* = 29)			
	Baseline	Post-operative week 2	Post-operative week 6	*p*-value^*^	Baseline	Post-operative week 2	Post-operative week 6	*p*-value^*^	Baseline	Post-operative week 2	Post-operative week 6	*p*-value^*^
Shoulder flexion AROM^+^(°)	160 (150–170)	155 (150–165)	160 (146–170)	0.775	165 (150–170)	159 (153–170)	160 (145–170)	0.551	158 (148–166)	155 (149–160)	160 (148–170)	0.319
Shoulder abduction AROM^+^(°)	157 (150–171)	159 (143–169)	175 (155–180)	**0.016**	160 (152–173)	165 (146–171)	175 (170–180)	0.443	154 (147–165)	159 (143–165)	172 (155–180)	**0.014**
Pain score^+#^	1 (1–1)	1 (1–2)	1 (1–2)	0.054	1 (1–1)	2 (1–2)	1 (1–2)	0.102	1 (1–1)	1 (1–2)	1 (1–2)	0.198
Numbness score^+#^	1 (1–1)	1 (1–2)	1 (1–2)	0.072	1 (1–1)	2 (1–2)	2 (1–2)	0.166	1 (1–1)	1 (1–2)	1 (1–2)	0.248
QuickDASH disability score^+^	0 (0–4.03)	6.82 (2.27–13.64)	2.5 (0–8.52)	**0.027**	2.27 (0–2.5)	6.82 (2.27–15)	5.0 (2.27–9.09)	**0.010**	0 (0–5.0)	6.82 (2.27–13.64)	2.27 (0–6.82)	0.396

*ALND* Axillary lymph node dissection, *AROM* Active range of motion

^+^All of the study variables in this table are presented in median (IQR) unless otherwise specified

^*^p-value is obtained by performing a Wilcoxon-signed rank test comparing baseline and post-operative week 6 values

^#^Pain score and numbness score are components of the QuickDASH questionnaire ranging from 1 to 5, where 1 represents none, 2 represents mild, 3 represents moderate, 4 represents severe and 5 represents extreme

**Fig. 3:**
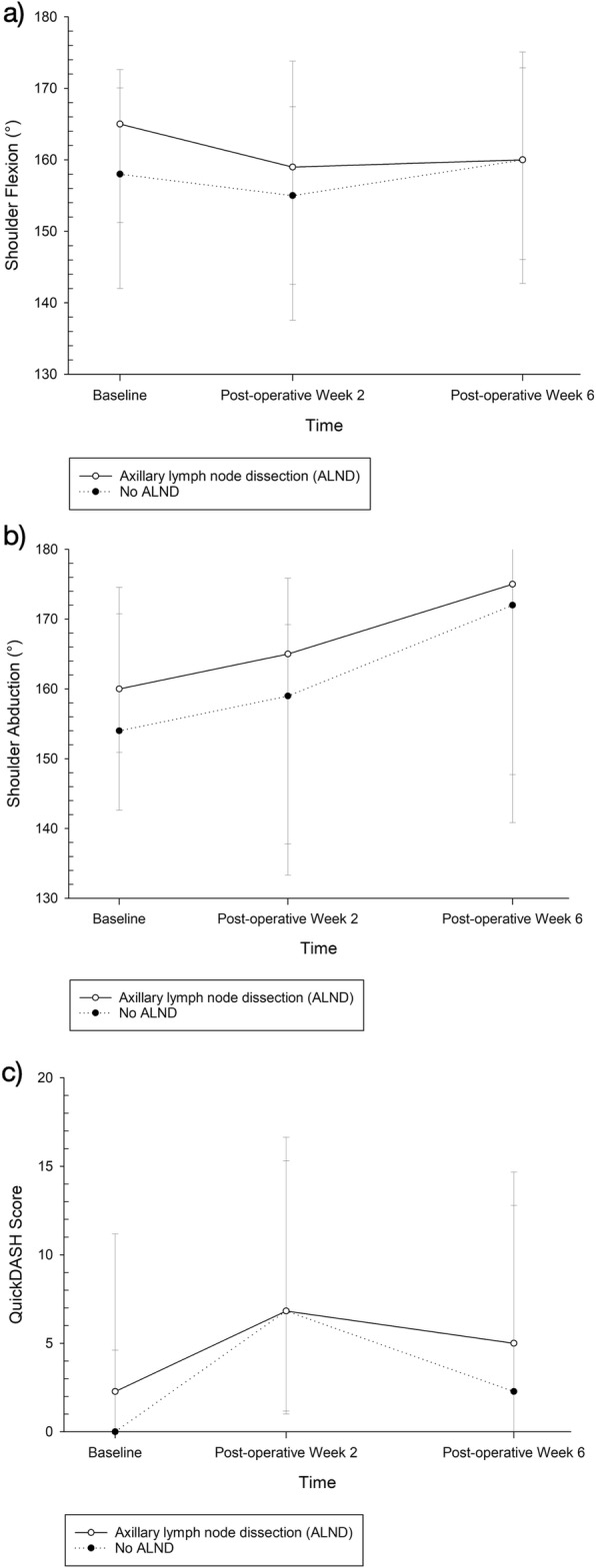
Changes in (**a**) shoulder flexion (**b**) shoulder abduction and (**c**) quickDASH disability score at baseline, post-operative week 2 and post-operative week 6 in the axillary lymph node dissection (ALND) and no ALND subgroup

Subgroup analysis of patients with ALND demonstrated significantly worse QuickDASH disability score at post-operative week 6 compared to baseline (*p* = 0.010) (Table 3). However, patients with only sentinel lymph node biopsy (SLNB) without ALND had comparable QuickDASH disability score at post-operative week 6 compared to baseline (*p* = 0.396). Figure 3a and b and c are pictorial representations of the change in shoulder flexion, abduction and quickDASH disability score in the ALND versus no ALND subgroup.

Table 4 summarises the upper limb function of patients with wide local excision with SLNB compared to mastectomy with SLNB. Shoulder flexion and abduction at post-operative week 2 is significantly lower in the group with mastectomy (*p* = 0.018 and *p* = 0.012 respectively), and also at post-operative week 6 (*p* = 0.019 and *p* = 0.008 respectively). Table 5 summarises the upper limb function of patients with wide local excision with ALND compared to mastectomy with ALND. Shoulder flexion in the mastectomy subgroup is significantly lower at post-operative week 6.

**Table 4:** Comparison of wide local excision (WLE) with sentinel lymph node biopsy (SLNB) versus mastectomy with SLNB

	Baseline			Post-operative week 2			Post-operative week 6		
	WLE + SLNB (*n* = 16)	Mastectomy + SLNB (*n* = 27)	*p*-value^*^	WLE + SLNB (*n* = 16)	Mastectomy + SLNB (*n* = 27)	*p*-value^*^	WLE + SLNB (*n* = 16)	Mastectomy + SLNB (*n* = 27)	*p*-value^*^
Shoulder flexion AROM^+^(°)	166 (151–171)	158 (148–165)	0.127	160 (155–167)	154 (140–159)	**0.018**	170 (160–174)	155 (142–170)	**0.019**
Shoulder abduction AROM^+^(°)	165 (152–173)	154 (145–165)	0.173	165 (159–170)	148 (133–167)	**0.012**	180 (175–180)	170 (145–180)	**0.008**
Pain score^+#^	1 (1–1)	1 (1–1)	0.583	1 (1–2)	1 (1–2)	0.452	1 (1–2)	1 (1–2)	0.771
Numbness score^+#^	1 (1–2)	1 (1–1)	0.442	1 (1–2)	1 (1–2)	0.103	1 (1–2)	2 (1–2)	**0.040**
QuickDASH disability score^+^	2.27 (0–5)	0 (0–2.5)	0.493	4.77 (0–13.07)	6.81 (2.5–13.6)	0.158	4.77 (0–8.52)	2.27 (0–9.09)	0.699

*AROM* Active range of motion, *WLE* Wide local excision

^+^All of the study variables in this table are presented in median (IQR) unless otherwise specified

^*^p-value is obtained by performing a Mann-Whitney U test comparing between the two groups at a specific time interval (baseline, post-operative week 2 or post-operative week 6)

^#^Pain score and numbness score are components of the QuickDASH questionnaire ranging from 1 to 5, where 1 represents none, 2 represents mild, 3 represents moderate, 4 represents severe and 5 represents extreme

**Table 5:** Comparison of wide local excision (WLE) with axillary lymph node dissection (ALND) versus mastectomy with ALND

	Baseline			Post-operative week 2			Post-operative week 6		
	WLE + ALND (*n* = 5)	Mastectomy + ALND (*n* = 10)	*p*-value^*^	WLE + ALND (*n* = 5)	Mastectomy + ALND (*n* = 10)	*p*-value^*^	WLE + ALND (*n* = 5)	Mastectomy + ALND (*n* = 10)	*p*-value^*^
Shoulder flexion AROM^+^(°)	166 (153–174)	163 (150–170)	0.679	165 (160–172)	155 (145–170)	0.254	170 (165–178)	155 (142–161)	**0.019**
Shoulder abduction AROM^+^(°)	171 (152–174)	160 (154–174)	0.953	170 (166–176)	148 (121–170)	0.055	180 (173–180)	175 (130–180)	0.310
Pain score^+#^	1 (1–1.5)	1 (1–1)	0.768	1 (1–2)	2 (1–2)	0.513	1 (1–1.5)	1.5 (1–2)	0.371
Numbness score^+#^	1 (1–2)	1 (1–1.3)	0.679	2 (1–2)	2 (1–2)	0.679	1 (1–1.5)	2 (1.8–2)	0.055
QuickDASH disability score^+^	2.27 (0–3.64)	1.14 (0–3.01)	0.953	5.00 (1.14–15.00)	8.41 (2.44–14.60)	0.679	5.00 (1.14–7.95)	5.68 (2.27–15.23)	0.513

*AROM* Active range of motion, *ALND* Axillary lymph node dissection

^+^All of the study variables in this table are presented in median (IQR) unless otherwise specified

^*^p-value is obtained by performing a Mann-Whitney U test comparing between the two groups at a specific time interval (baseline, post-operative week 2 or post-operative week 6)

^#^Pain score and numbness score are components of the QuickDASH questionnaire ranging from 1 to 5, where 1 represents none, 2 represents mild, 3 represents moderate, 4 represents severe and 5 represents extreme

## Discussion

There has been increasing interest in functional outcomes after cancer surgery in the surgical community [16]. This is especially relevant for surgeries with high fidelity, excellent surgical and oncological outcomes such as breast cancer surgery [3]. In this group of patients, the next focus is to improve on overall holistic care and to ensure that they return back to baseline function. Functional decline of the upper limb after major breast surgery has been reported not infrequently and this is one of the first studies to our knowledge to examine this phenomenon in the Asian population [4–7].

Our unit adopts an early and aggressive rehabilitation regime which encourages patients to start mobilizing and perform upper limb exercises in their homes from the first post-operative day. Objectively, this pilot study demonstrated return to baseline function in shoulder AROM at 6 weeks post-operative. Gosselink et al. who described good functional recovery at 3 months also adopted a similar early rehabilitation regime from post-operative day 2 in their centre [10]. This is in contrast to other studies who reported significant residual functional impairment whose rehabilitation regime are only commenced at 2 weeks to 1 month post-operative [11, 17]. Delayed stretching and rehabilitation may be less effective as scar tissue become denser and are less receptive to short-term stretching [18, 19]. However, it is also worth noting that a study by Scaffidi et al. which compared early and delayed rehabilitation showed no difference in shoulder impairment at post-operative day 60 [11].

Interestingly, our study showed an improvement in shoulder abduction at post-operative week 6 compared to baseline (post-operative week 6 AROM 175° vs baseline AROM 157°, *p* = 0.016). This phenomenon has been demonstrated in another study by Beurskens et al. who demonstrated an increase in shoulder flexion and abduction AROM from baseline after physiotherapy post breast surgery and axillary dissection [9]. We postulate that some patients may have impaired upper limb function pre-operatively either from pain or axilla lymph node disease that benefited from surgery and an aggressive post-operative rehabilitation regime.

Despite demonstrating objective return of shoulder AROM back to baseline, our study found a significantly worse QuickDASH disability score at post-operative week 6 (median score 2.5, IQR 0–8.52, *p* = 0.027) compared to baseline. We postulate that this phenomenon may be contributed by the presence of pain and numbness in the upper limb despite regaining AROM. A possible consideration will be to extend the duration of rehabilitation with an existing study by Beurskens et al. reporting a significant improvement in pain following physiotherapy at 3 months [9]. However in the era where value based care is a buzz word in Singapore, cost effectiveness of such an approach is debatable.

The question of which is the best measurement of upper limb function is still unanswered as various studies have reported heterogenous upper limb outcome measures which include shoulder mobility, pain intensity, presence of lymphedema and assessment of activities of daily living [10, 20]. We used the quickDASH disability score for the study as it is a simple patient reported 11-point questionnaire which assesses subjective physical function, important symptoms such as pain and numbness and its impact on daily activities [15]. This may be more holistic compared to earlier studies which solely evaluate symptoms without assessment of impact on daily activities. We believe the use of both AROM and quickDASH disability score in our study allows a standardized holistic assessment of the overall function of the patient.

Subgroup analysis of patients with and without ALND (Table 3) demonstrated worse QuickDASH disability score at post-operative week 6 compared to baseline only for patients with ALND. This is consistent with findings from Western studies as ALND is a known predictor of short-term upper limb morbidity [7, 21]. However, our subgroup analysis showed superior results for patients who only had SLNB without ALND as compared to the study by Rietman et al. on 204 patients, which demonstrated significant decrease in shoulder AROM at post-operative week 6 following SLNB [21]. Similarly, we believe that early rehabilitation may be the contributing factor to our favourable results.

Our study also demonstrated worse upper limb function at 6 weeks for mastectomy compared to WLE regardless of whether SLNB or axillary clearance was performed. This finding is consistent with a systematic review by Lee et al. who demonstrated mastectomy as a risk factor for reduced shoulder range of motion compared to breast-conserving surgery (OR 5.67, 95% CI 1.03–31.16) [22]. This information deserves to be highlighted to the patient during pre-operative counselling and taken into consideration during informed consent since the outcome impacts on function and quality of life. This also reinforces the importance of rehabilitation after mastectomy especially those who received ALND, as the risk of lymphoedema can be minimized which improves eventual upper limb function.

One of the limitations is the relatively small sample size in this pilot study. However, it has been well-described in literature that a minimum of 10–12 patients in a pilot study provides the ability to test a hypothesis [23, 24]. We are also unable to enforce compliance as the exercises were performed in the patient’s home. However, prior to discharge, the physiotherapist will conduct a dedicated session with the aid of pamphlets for the patient and educate them on the importance of following the exercise regime strictly and will further reinforce this information at the 2 weeks follow-up appointment. Lastly, the follow-up period in our study is relatively short and it is likely that patients’ disability score may continue to improve after the 6 weeks.

## Conclusions

This pilot study in an Asian cohort found that patients were able to regain AROM of shoulder after major breast surgery at post-operative week 6 but had a worse QuickDASH disability score, especially in the subgroup with ALND. Aggressive and early rehabilitation should be encouraged. However, a longer follow-up is required to evaluate long term functional outcomes.

## Supplementary information

**Additional file 1: Appendix A.** Measurement of shoulder range of motion. **Figure A.1**. Pictorial representation of measurement of shoulder range of motion using a goniometer. Written consent has been obtained from both of the people included in the figure. **Table A.1**. Measurement of shoulder range of motion using a goniometer. **Appendix B**: Details of the QuickDASH Questionnaire. **Table B.1.** Components of the QuickDASH Questionnaire. **Table B.2.** Breakdown of components of the QuickDASH questionnaire. **Appendix C**: Physiotherapy exercises. **Table C.1**. List of physiotherapy exercises prescribed for patients post-major breast surgery. **Table C.2.** Post-operative rehabilitation protocol

## Data Availability

The datasets used and/or analysed during the current study are available from the corresponding author on reasonable request.

## Abbreviations

ALND: Axillary lymph node dissection
AROM: Active range of motion
WLE: Wide local excision
