# Supplementary material for: Measuring upper limb function and patient reported outcomes after major breast cancer surgery: a pilot study in an Asian cohort
Source: BMC Surg. 2020 May 19;20:108. doi: 10.1186/s12893-020-00773-0 (PMC7236525; doi:10.1186/s12893-020-00773-0)
Supplement: Supplementary file 1 — Additional file 1: Appendix A. Measurement of shoulder range of motion. Figure A.1. Pictorial representation of measurement of shoulder range of motion using a goniometer. Written consent has been obtained from both of the people included in the figure. Table A.1. Measurement of shoulder range of motion using a goniometer. Appendix B: Details of the QuickDASH Questionnaire. Table B.1. Components of the QuickDASH Questionnaire. Table B.2. Breakdown of components of the QuickDASH questionnaire. Appendix C: Physiotherapy exercises. Table C.1. List of physiotherapy exercises prescribed for patients post-major breast surgery. Table C.2. Post-operative rehabilitation protocol [file 12893_2020_773_MOESM1_ESM.docx]

**Supplementary Material**

**Appendix A: Measurement of shoulder range of motion**

Figure A.1: Pictorial representation of measurement of shoulder range of motion using a goniometer. Written consent has been obtained from both of the people included in the figure.


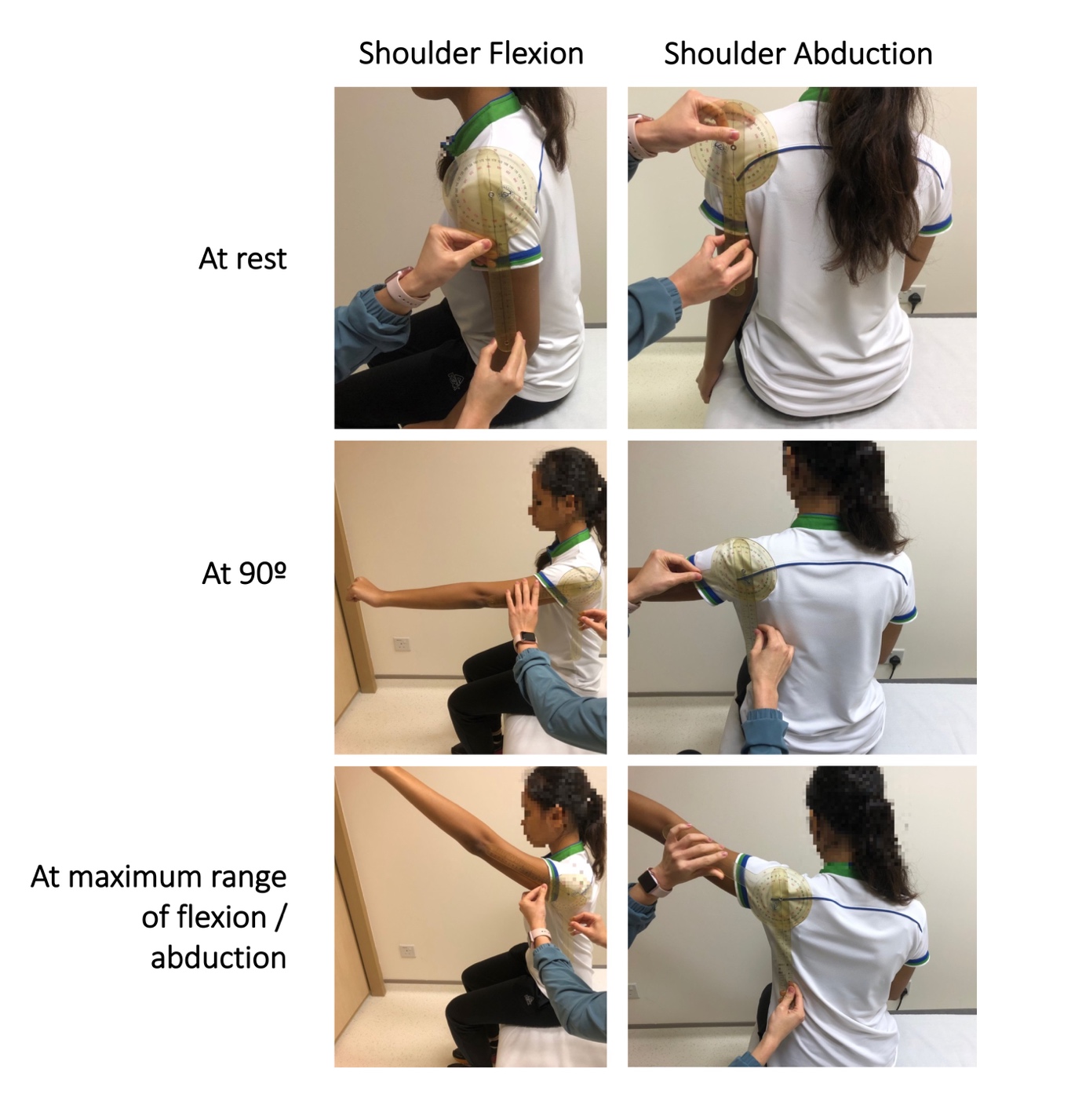


Table A.1: Measurement of shoulder range of motion using a goniometer

| Shoulder flexion | | | |
| --- | --- | --- | --- |
| Test position | Goniometer alignment | Testing | Possible mistakes/ compensations |
| - Use 12’ Goniometer - Sitting in chair - Feet on floor - Back unsupported - Arms at side | - Axis – center of humeral head near acromion process - Stationary arm – parallel to mid-axillary line - Moving arm – aligned with midline of humerus (lateral epicondyle) | - Have individual raise arm forward and overhead as high as possible with thumbs leading - Fixed arm of goniometer stays in place, movable arm moves with humerus | - Arching at lumbar or thoracic spine - Shoulder hitching - Bending at elbow - Measure angle at point of compensation |
| Shoulder abduction | | | |
| Test position | Goniometer alignment | Testing | Possible mistakes/compensations |
| - Use 12’ Goniometer - Sitting in chair - Feet on floor - Back unsupported - Arms at side | - Measure from behind - Axis – posterior aspect of glenohumeral joint acromion process just inferior to acromion - Stationary arm – parallel to trunk - Moving arm – aligned with midline of humerus (lateral epicondyle) | - Have individual raise arm to the as high as possible with thumbs leading - Fixed arm of goniometer stays in place, movable arm moves with humerus | - Sideways lean of trunk - Shoulder hitching - Shoulder going into forward flexion - Measure angle at point of compensation |

**Appendix B: Details of the QuickDASH Questionnaire**

Table B.1: Components of the QuickDASH Questionnaire

| Category | Questions | Interpretation |
| --- | --- | --- |
| General Questions | Please rate the difficulty of performing the following actions:   1. Open a tight or new jar 2. Do heavy household chores (eg wash walls, floors) 3. Carry a shopping bag or briefcase 4. Wash your back 5. Use a knife to cut food 6. Recreational activities in which you take some force or impact through your arm, shoulder or hand (eg. golf, hammering, tennis etc) | - 1 = No Difficulty - 2 = Mild Difficulty - 3 = Moderate Difficulty - 4 = Severe Difficulty - 5 = Unable |
|  | Please rate the extent in which you have been affected:   1. During the past week, to what extent has your arm, shoulder or hand problem interfered with your normal social activities with family, friends, neighbours or groups? 2. During your past week, were you limited in your work or other regular daily activities as a result of your arm, shoulder or hand problem? | - 1 = Not at all - 2 = Slightly - 3 = Moderately - 4 = Quite a bit - 5 = Extremely |
|  | Please rate the severity of the following symptoms in the last week:   1. Arm, shoulder or hand pain 2. Tingling (pins and needles) in your arm, shoulder or hand 3. During the past week, how much difficulty have you had sleeping because of the pain in your arm, shoulder or hand? | - 1= None - 2 = Mild - 3 = Moderate - 4 = Severe - 5 = Extreme |
| **QuickDASH Disability Score**  **Score = [(sum of n responses)/n – 1] x 25, where n is equal to the number of completed responses.**  Note: At least 10 of the 11 items must be completed for a score to be calculated. The assigned values for all completed responses are simply summed and averaged, producing a score out of five. This value is then transformed to a score out of 100 by subtracting one and multiplying by 25. This transformation is done to make the score easier to compare to other measures scaled on a 0-100 scale. A higher score indicates greater disability. | | |

Table B.2: Breakdown of components of the QuickDASH questionnaire.

| Question  No. | Baseline | Post-operative week 2 | Post-operative week 6 |
| --- | --- | --- | --- |
| 1 | 1.34 ± 0.91 | 1.18 ± 0.66 | 1.25 ± 0.69 |
| 2 | 1.05 ± 0.30 | 1.44 ± 0.91 | 1.16 ± 0.43 |
| 3 | 1.02 ± 0.15 | 1.07 ± 0.26 | 1.23 ± 0.71 |
| 4 | 1.14 ± 0.64 | 1.27 ± 0.50 | 1.09 ± 0.36 |
| 5 | 1.00 ± 0.00 | 1.02 ± 0.15 | 1.02 ± 0.15 |
| 6 | 1.00 ± 0.00 | 1.36 ± 0.83 | 1.21 ± 0.73 |
| 7 | 1.11 ± 0.44 | 1.48 ± 0.51 | 1.16 ± 0.37 |
| 8 | 1.09 ± 0.36 | 1.59 ± 0.73 | 1.30 ± 0.55 |
| 9 | 1.14 ± 0.46 | 1.50 ± 0.55 | 1.39 ± 0.58 |
| 10 | 1.27 ± 0.54 | 1.51 ± 0.63 | 1.50 ± 0.63 |
| 11 | 1.02 ± 0.15 | 1.30 ± 0.46 | 1.32 ± 0.74 |

All values are provided in mean ± standard deviation

Please refer to Table B.1 for the questions and respective interpretation

**Appendix C: Physiotherapy exercises**

Table C.1: List of physiotherapy exercises prescribed for patients post-major breast surgery

|  | **Instructions** |
| --- | --- |
| **Shoulder Rotation** | - Keep both shoulders relaxed. Roll your shoulders clockwise. - Change direction. |
| **Assisted Shoulder Elevation** | - Hold your affected arm with the other arm. - Assist to lift your arm up as far as possible, let your thumb lead the way. |
| **Posterior Capsule Stretch** | - Stretch your affected arm across the shoulder by pushing it at the elbow with the other arm. |
| **Shoulder Pendulum** | - Stand leaning on a table with your good hand. - Relax your arm on the operated side. Swing your arm as if you are drawing a circle on the floor. - Change direction. |
| **Hand Behind Back** | - Stand upright. - Hold your affected arm behind your back (at waist level) with the other hand. - Pull your affected arm across the body and upwards. |
| **Wall Climb** | - Stand near a wall. - Slowly ‘walk’ your fingers up the wall, so that you feel a stretch. - Hold for a few seconds. |

Table C.2: Post-operative rehabilitation protocol

| **Timeframe** | **Day 1 post-operative** | **Week 2 post-operative** | **Week 6 post-operative** |
| --- | --- | --- | --- |
| **Assessment** | - Assess wound site - Assess shoulder range of motion in flexion and abduction | - Assess wound site - Assess shoulder AROM - Assess upper limb circumference - Assess potential impairments as indicated (e.g. muscle length, neck AROM) | - Assess shoulder AROM in Flexion and Abduction as per study protocol - Conduct QuickDASH questionnaire as per study protocol |
| **Treatment** | - Shoulder range of motion exercises (refer below) - Encourage ambulation and deep breathing exercises - Advice to avoid heavy lifting 2-4 weeks, ensure proper skincare of affected arm | Treatment is dependent on assessment findings   - Shoulder ROM exercises - Manual techniques as indicated | Treatment is dependent on assessment findings   - Shoulder ROM exercises - Manual techniques as indicated - Shoulder strengthening exercises |
| **Follow up management** | - Date of next review is dependent on assessment findings and treatment outcomes - Refer to Outpatient Women’s health clinic for 2 weeks post-operative if patient has severe pain or does not have full range of shoulder movement - If full range of shoulder movement, next review is made for 6 weeks post-operative as per study protocol | - Date of next review is dependent on assessment findings and treatment outcomes - If full range of shoulder movement, next review is made for 6 weeks post-operative as per study protocol | - Date of next review is dependent on assessment findings and treatment outcomes - If full range of shoulder movement, no further appointment is made |
